# Resource Allocation in Multi-Carrier Multiplexed NOMA Cooperative System

**DOI:** 10.3390/s22166023

**Published:** 2022-08-12

**Authors:** Jie Zeng, Jiaying Sun, Yuxin Song, Jiajia Mei, Tiejun Lv, Shidong Zhou

**Affiliations:** 1 Beijing National Research Center for Information Science and Technology, Tsinghua University, Beijing 100084, China; 2 Department of Electronic Engineering, Tsinghua University, Beijing 100084, China; 3 State Key Laboratory of Wireless Mobile Communications, China Academy of Telecommunication Technology, Beijing 100084, China; 4 School of Communication and Information Engineering, Chongqing University of Posts and Telecommunications, Chongqing 400065, China; 5 The National Key Laboratory of Science and Technology on Communications, University of Electronic Science and Technology of China, Chengdu 611731, China; 6 School of Information and Communication Engineering, Beijing University of Posts and Telecommunications, Beijing 100876, China

**Keywords:** non-orthogonal multiple access, cooperative communication, dynamic group matching, resource allocation, ultra-dense networks

## Abstract

Non-orthogonal multiple access (NOMA) cooperative communication technology can combine the advantages of NOMA and cooperative communication, providing high spectrum efficiency and increasing user coverage for next-generation wireless systems. However, the research on NOMA cooperative communication technology is still in a preliminary stage and has mainly concentrated on the scenario of fewer users. This paper focuses on a user-centered NOMA collaboration system in an ultra-dense network, and it constructs a resource allocation optimization problem to meet the demands of each user. Then, this paper decomposes the optimization problem into two subproblems; one is the grouping match among multiple relays and users, and the other is jointly allocating power and subcarrier resources. Accordingly, a dynamic packet matching algorithm based on Gale–Shapley and an iterative algorithm based on the difference of convex functions programing are proposed. Compared with existing schemes, the proposed algorithms can improve system throughput while ensuring the quality of service of users.

## 1. Introduction

It is predictable that with the expansion in the Internet of Things (IoT) and the development of communication, a large number of wireless connections and huge data traffic will pose challenges for next-generation wireless systems [1]. The demand for spectrum efficiency and network capacity has grown rapidly [2]; since orthogonal resources are limited, traditional orthogonal multiple access (OMA) has difficulty meeting the multiple UEs demands [3,4]. Recently, due to the superior spectral efficiency, non-orthogonal multiple access (NOMA) has attracted tremendous attention in industry and academia [5]. Compared with traditional OAM, NOMA can reuse non-orthogonal superposition and then assume large-scale connectivity [6,7].

Previous research on single-carrier NOMA technology is relatively complete, and performance evaluations at the link and system levels have proven that NOMA has a better transmission rate and lower error rate than OMA systems [8]. For multi-carrier NOMA technology, the research is not sufficient and has mainly focused on the typical sparse code multiple access (SCMA) and pattern division multiple access (PDMA) technology. Research on SCMA technology mainly focuses on codebook design, channel transmission rate assessment and receiver design [9,10,11,12]. Research on PDMA technology has primarily focused on pattern design, interruption probability analysis and receiver design [13,14,15].

Cooperative communication is an important method for combating channel fading and path loss and for reducing the shadow effect [16,17]. It was first introduced in [18] and became an important component in the previous communication network. With the distributed transmission of relay at the transmitting site and the combination of signals at the receiving site, cooperative communication technology can obtain a cooperative diversity gain similar to the spatial diversity gain of the multiple input multiple output (MIMO) [19]. Because of the above feature, cooperative communication is widely used in IoT scenarios where the size of massive UEs is small and difficult to implement multiple antennas [20,21].

NOMA cooperative communication technology was introduced in [22]. It can combine the advantages of NOMA and cooperative communication to meet the demands of next-generation communication. However, there have been few studies addressing the combination of the two technologies, especially on NOMA multi-carrier cooperative communication technology.

Multi-carrier multiplexed NOMA cooperative technology can effectively obtain multiplexing diversity gain and improve system performance [23]. Luo et al. [24] studied the resource allocation optimization of an SCMA cooperative system, and the optimization was carried out with the weighted sum of power, codebook and subcarrier pairing as the alternative objective. Han et al. [25] studied the method of self-interference mitigation for an SCMA cooperative system with a large-scale transceiver antenna. Tang et al. [26] proposed an uplink PDMA collaboration system with a half-duplex decoder and relay, and they analyzed the interrupt performance. Tang et al. [27] analyzed the outage probability of a downlink PDMA collaboration system with decoding and forwarding of a half-duplex relay. However, in [26,27], the derived closed-form expression of interrupt probability was simplified to only the scenario with three users and two carriers while not considering the return link discontinuity. The more general interrupt probability expression and the full-duplex scenario still need to be studied and analyzed. In addition, Sun et al. [28] studied the multi-carrier NOMA cooperative system, which adopts a full-duplex base station for upstream and downstream simultaneous transmission, but the resource allocation optimization algorithm also assumes that the stack number of upstream or downstream users on each subcarrier does not exceed 2.

There are still several problems in the previous research studies. First, the theoretical boundaries of capacity and outage probability in a general scenario are not clear. Second, scheduling and resource allocation under multi-user and multi-relay with multi-carrier are extended to larger dimensions. Furthermore, effective multi-dimensional constellations, such as those in SCMA and PDMA, are more difficult to design and optimize than single-dimensional power segmentation. Finally, the widespread deployment of multi-carrier NOMA in conjunction with existing orthogonal frequency division multiple access lacks viable applications and validation.

In this paper, we studied multi-carrier multiplexed NOMA cooperative technology in a super-dense network, and the main contributions of this paper are listed below.

- We designed a user-centered multi-carrier multiplexed NOMA cooperative system that can fully combine the advantages of multi-carrier NOMA and cooperative communication technology to meet the abundant UE demands.
- We constructed a problem to optimize throughput while ensuring multiple users’ demands and decomposed it into two subproblems. Then, we proposed the corresponding dynamic grouping matching algorithm and iterative algorithm based on the difference of convex functions programing (DCP) to solve them.
- Simulations were used to verify the effectiveness of the proposed NOMA cooperative network framework and the corresponding algorithms. Compared with two existing schemes, combining the dynamic grouping matching algorithm with an iterative algorithm improved system throughput while ensuring user quality of service (QoS).

The remainder of the paper is organized as follows. Section 2 describes the system model, including the signaling model and the throughput model. In Section 3, the problem is formulated as an optimization problem. In Section 4, we propose the resource allocation algorithm of the cooperative network. Section 5 presents the simulation results, which prove the effectiveness of the proposed algorithm. Finally, the conclusions are drawn in Section 6.

## 2. System Model

**Figure 1:**
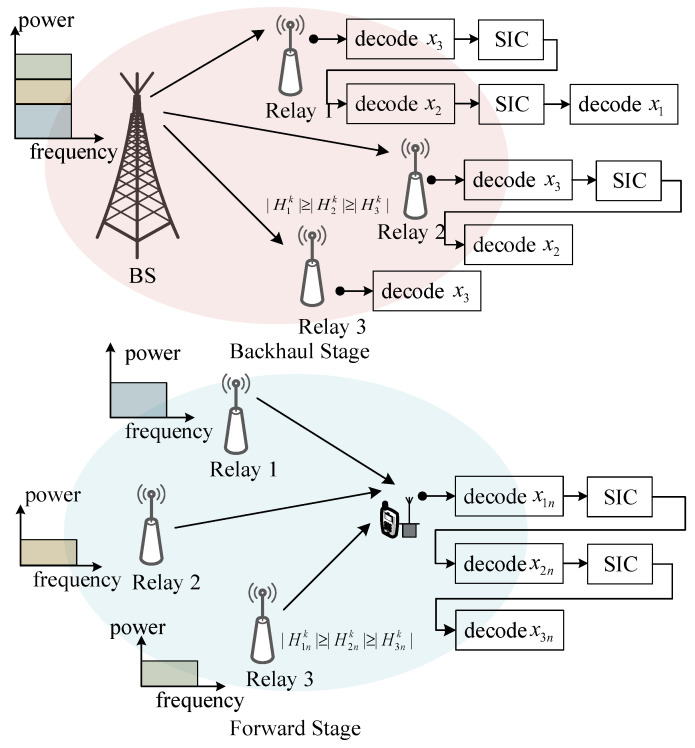
An illustration of the two-stage transmission based on NOMA.

In this section, we describe the downlink NOMA-based cooperative network setting [29] consisting of a base station (BS), *M* relays, and *N* UEs, as shown in Figure 1. Each node is equipped with a transmit antenna and a receive antenna. The system frequency band is divided into *K* subcarriers. The signals of different UEs or different packets of a UE can be superposed in one subcarrier to transmit simultaneously. In addition, all the relays are connected to the BS in the backhaul stage. We assume that the UEs and relays follow two independent Poisson point processes (PPPs) with the densities of $\lambda_{u}$ and $\lambda_{r}$, respectively. The notions about the system model in this section are listed in Table 1.

The BS can support the backhaul stage for relays and provide access services for UEs. In this paper, the UEs are assumed to be served dynamically via dense relays. The BS and the relays share the same frequency band, and the relays work in the time division duplex (TDD) mode. The signals passed into the backhaul stage and forward stage do not affect each other. At the backhaul stage, the signal is transmitted from the BS to the relays, and then, the relays decode the information of users and transmit it to corresponding users during the forward stage. Therefore, the downlink transmission can be divided into two processes, backhaul transmission and forward transmission, as illustrated in Figure 1.

### 2.1. Signaling Model

In the forward stage, we take the *n*th UE as an example to illustrate the signals in the downlink cooperative network, n∈{1,2,…,N}. The inter-group interference can be avoided by properly grouping relays and allocating subcarriers [30]. We use $x_{mn}^{k}$ to denote the signal, transmitted from the *m*th relay to the *n*th UE on the *k*th subcarrier. The transit power of $x_{mn}^{k}$ is $p_{mn}^{k}$. Meanwhile, $h_{mn}^{k}$ and $\varpi_{mn}^{}$ are the small-scale and large-scale fading channel coefficients from the *m*th relay to the *n*th UE on the *k*th subcarrier, respectively. The channels of the forward and backhaul stages are independent Rayleigh fading channels, and the path loss exponent is α. $\varpi_{mn}^{}=d_{mn}^{-\alpha }$, where $d_{mn}$ denotes the distance from the *m*th relay to the *n*th UE. Then, the signal received on the *k*th subcarrier of the *n*th UE can be written as

(1) $$y_{n}^{A,k}=\sqrt{p_{mn}^{k}\varpi_{mn}^{}}h_{mn}^{k}x_{mn}^{k}+\sum_{l\neq m}^{M}\sqrt{p_{ln}^{k}\varpi_{mn}^{}}h_{ln}^{k}x_{ln}^{k}+z_{n}^{k},$$

where $z_{n}^{k}$ is the AWGN at the receiver of the *n*th UE on the *k*th subcarrier with mean zero and variance $\sigma^{2}$.

In the backhaul stage, we take the *m*th relay (m∈{1,2,…,M}) as an example to analyze the backhaul stage signals in the considered downlink cooperative network. Suppose that the signal transmitted from the BS to the *m*th relay on the *k*th subcarrier is $x_{m}^{k}$, and the power of the signal is $q_{m}^{k}$. Additionally, $h_{m}^{k}$ and $\varpi_{m}$ denote the small-scale and large-scale channel coefficients from the BS to the *m*th relay on the *k*th subcarrier. Then, the received signal on the *k*th subcarrier of the *m*th relay can be written as

(2) $$y_{m}^{B,k}=\sqrt{q_{m}^{k}\varpi_{m}}h_{m}^{k}x_{m}^{k}+\underset{\mathrm{interference}\;\mathrm{of}\;\mathrm{other}\;\mathrm{relays}\;}{\underset{︸}{h_{m}^{k}\sum_{l\neq m}^{M}\sqrt{q_{l}^{k}\varpi_{l}}x_{l}^{k}}}+z_{m}^{k},$$

where $z_{m}^{k}$ denotes the additive white Gaussian noise (AWGN) with mean zero and variance $\sigma^{2}$ at the receiver of the *m*th relay on the *k*th subcarrier.

### 2.2. Throughput Model

Since the transmission process is divided into two stages, the throughput is analyzed separately at the two stages.

In the forward stage, since the successive interference cancellation (SIC) technique is applied at the receivers to decode the signals from different relays, we assume that the channel coefficients meet $|H_{1n}^{k}\left|⩾\right|H_{2n}^{k}\left|⩾\ldots ⩾\right|H_{Mn}^{k}|$. $H_{mn}^{k}=\varpi_{m}^{}h_{mn}^{k}$ represents the channel coefficient between the *n*th UE and the *m*th relay on the *k*th subcarrier. Then, the decoding order is consistent with the relay indexes. Thus, the received signal-to-interference-plus-noise ratio (SINR) of the *n*th UE served by the *m*th relay on the *k*th subcarrier is written as

(3) $$SINR_{mn}^{A,k}=\frac{\varpi_{mn}^{}p_{mn}^{k}|h_{mn}^{k}{|}^{2}}{\sigma^{2}+\sum_{l=m+1}^{M}|h_{ln}^{k}{|}^{2}\varpi_{ln}^{}p_{ln}^{k}}.$$

The corresponding throughput of the *n*th UE is given by

(4) $${\tilde{R}}_{n}^{A}=\sum_{k=1}^{K}\sum_{m=1}^{M}c_{mn}^{k}\beta^{k}\log_{2}\left(1+SINR_{mn}^{A,k}\right).$$

In the backhaul stage, without loss of generality, we assume that the channel coefficients yield $|H_{1}^{k}\left|⩾\right|H_{2}^{k}\left|⩾\ldots ⩾\right|H_{M}^{k}|$. Here, $H_{m}^{k}=\sqrt{\varpi_{m}^{}}h_{m}^{k}$ represents the channel coefficient between the BS and the *m*th relay on the *k*th subcarrier. Then, the decoding is carried out in the reverse order of the relay indexes. The SINR of the *m*th relay on the *k*th subcarrier is given by

(5) $$SINR_{m}^{B,k}=\frac{\varpi_{m}^{}q_{m}^{k}|h_{m}^{k}{|}^{2}}{\sigma^{2}+\varpi_{m}^{}|h_{m}^{k}{|}^{2}\sum_{l=1}^{m-1}q_{l}^{k}}.$$

Correspondingly, the backhaul throughput for the *n*th UE can be given by

(6) $${\tilde{R}}_{n}^{B}=\sum_{k=1}^{K}\sum_{m=1}^{M}c_{mn}^{k}\left(1-\beta^{k}\right)\log_{2}\left(1+SINR_{m}^{B,k}\right),$$

where $c_{mn}^{k}$ indicates whether the *n*th UE is served by the *m*th relay on the *k*th subcarrier or not, and $c_{mn}^{k}\in \left\{0,1\right\}$. If $c_{mn}^{k}$=1, the *n*th UE is served by the *m*th relay on the *k*th subcarrier. $\beta^{k}$ ($0⩽\beta^{k}⩽1$) denotes the proportion of a time slot occupied by the forward stage on the *k*th subcarrier, and $1-\beta^{k}$ is the proportion of the time slot used for backhaul.

Since the signal needs to be transmitted to the relays first and then forwarded to the UEs, the system throughput is given by

(7) $$\tilde{R}=\sum_{n=1}^{N}{\tilde{R}}_{n}^{A},{\tilde{R}}_{n}^{A}\leq {\tilde{R}}_{n}^{B}.$$

## 3. Problem Formulation

In this section, we maximize the system throughput under QoS constraints. Because the SIC technique is applied at the receiver, the complexity of the receiver grows with the number of superposed signals on a subcarrier. We assume that each UE can be served by up to *Q* relays on a subcarrier to harness the complexity of the receiver at the UEs. The constraints are written as

(8) $$\begin{array}{cc} C1\;\;\; & (a):c_{mn}^{k}\in \left\{0,1\right\},\forall m,n,k\\ \; & (b):\sum_{m=1}^{M}c_{mn}^{k}⩽Q,\forall n,k. \end{array}$$

Apart from the overall power constraints of the system, the power allocation in the NOMA system needs to satisfy the threshold for SIC decoding at the receiver (cf., the OMA system). Therefore, constraints *C*2 and *C*3 are given by

(9) $$\begin{array}{cc} C2\;\;\; & \left(a\right):\sum_{k=1}^{K}p_{mn}^{k}⩽P_{m,\max}^{},\forall m\\ & \left(b\right):\sum_{m=1}^{M}\sum_{k=1}^{K}q_{m}^{k}⩽P_{\max}^{\mathrm{BS}}, \end{array}$$

and

(10) $$\begin{array}{cc} C3\;\;\; & \left(a\right):\varpi_{mn}p_{mn}^{k}|h_{mn}^{k}{|}^{2}-\sum_{q=m+1}^{M}\varpi_{qn}|h_{qn}^{k}{|}^{2}c_{qn}^{k}p_{qn}^{k}\\ & ⩾p_{\mathrm{thr}},\forall m,n,k\;\\ \; & \left(b\right):\varpi_{m}|h_{m}^{k}{|}^{2}\left(q_{m}^{k}-\sum_{l=1}^{m-1}q_{l}^{k}\right)⩾p_{\mathrm{thr}},\forall m,k, \end{array}$$

where $P_{\max}^{\mathrm{BS}}$ and $P_{m,\max}^{}$ denote the maximum available powers of the BS and the *m*th relay, respectively, and $p_{\mathrm{thr}}$ is the decoding power threshold for the SIC receiver.

In terms of the QoS of each UE, we consider

(11) $$C4:{\hat{R}}_{n}^{\mathrm{target}}⩽{\tilde{R}}_{n}^{A}⩽{\tilde{R}}_{n}^{B},\forall n,$$

where ${\hat{R}}_{n}^{\mathrm{target}}$ denotes the target data rate of the *n*th UE. Additionally, the time slot assignment coefficient between the access stage and backhaul stage on an arbitrary *k*th subcarrier needs to meet

(12) $$C5:0⩽\beta^{k}⩽1,\forall k.$$

Therefore, the optimization problem can be formulated as

(13) $$\begin{array}{cc} \; & \underset{c,\mathrm{fi},p,q}{\mathrm{maximize}\;}\;\;\tilde{R}\left(c,\mathrm{fi},p,q\right)=\sum_{n=1}^{N}{\tilde{R}}_{n}^{A}\;\\ \; & s.t.\;\;C1,C2,C3,C4,C5, \end{array}$$

where $q\in R_{+}^{KM\times 1}$ and $p\in R_{+}^{NKM\times 1}$ collect the power $q_{m}^{k}$ allocated on the BS and the power $p_{mn}^{k}$ allocated on the relays, respectively. $c\in Z^{NKM\times 1}$ and $\mathrm{fi}\in R^{K\times 1}$ collect the variables $c_{mn}^{k}$ and $\beta^{k}$, respectively.

## 4. Resource Allocation Algorithms

Problem (13) is a mixed integer non-linear programming problem. It is challenging to derive a global-optimal solution [31]. In this paper, a low-complexity suboptimal solution is developed in the presence of multiple relays and UEs. Problem (13) is divided into two subproblems. First, we apply a dynamic group matching algorithm to map each UE with relays. Then, an iterative algorithm is proposed based on the D.C. programming to achieve a suboptimal solution for the joint power and subcarrier allocation.

### 4.1. Dynamic Group Matching for UEs and Relays

The grouping process of relays and UEs is a matching process between each UE and a set of relays serving the UE. To maximize the system throughput, we apply a deferred-acceptance strategy from the Gale–Shapley algorithm to balance the two-side matching priority of the UEs and relays. Let Φ(m,n) represent the matched pair of the *m*th relay and the *n*th UE, and let Φ denote the set of matched pairs. |Φ(m,n)|=1 denotes that the *n*th UE is matched with the *m*th relay; otherwise, |Φ(m,n)|=0. We define an evaluation model of the pair between the *n*th UE and the *m*th relay as

(14) $$R_{mn}=\log_{2}\left(1+SINR_{mn}^{A}\right),$$

where

(15) $$SINR_{mn}^{A}=\frac{\varpi_{mn}^{}|h_{mn}^{k}{|}^{2}}{\sigma^{2}+\sum_{l=m+1}^{M}\varpi_{ln}^{}|h_{ln}^{k}{|}^{2}}.$$

With a two-sided competitive selection of the UEs and relays, each node has its matching priority list to match with others. We denote the matching priority sets of UEs and relays as

(16) $$\left\{MP\_UE\right\}=\left\{MP\_UE_{1},\ldots ,MP\_UE_{n},\ldots ,MP\_UE_{N}\right\},$$

(17) $$\left\{MP\_RE\right\}=\left\{MP\_RE_{1},\ldots ,MP\_RE_{m},\ldots ,MP\_RE_{M}\right\},$$

where $MP\_UE_{n}$ is the matching priority list that the *n*th UE matches with its nearby relays; similarly, $MP\_RE_{m}$ is the matching priority list of the nearby UEs that the *m*th relay can match with. They can be further represented as

(18) $$\begin{array}{cc} \; & MP\_UE_{n}\\ & =\{MP\_UE_{n}\left(1\right),\ldots ,MP\_UE_{n}\left(m_{n}\right)\ldots ,MP\_UE_{n}(M_{n})\}, \end{array}$$

(19) $$\begin{array}{cc} \; & MP\_RE_{m}\\ & =\{MP\_RE_{m}\left(1\right),\ldots ,MP\_RE_{m}\left(n_{m}\right)\ldots ,MP\_RE_{m}(N_{m})\}, \end{array}$$

where $M_{n}$ and $N_{m}$ are the number of relays near the *n*th UE and the number of UEs near the *m*th relay, respectively; $MP\_UE_{n}\left(m_{n}\right)$ denotes the relay whose matching priority of the *n*th UE is $m_{n}$, and $MP\_RE_{m}\left(n_{m}\right)$ denotes the UE whose matching priority of the *m*th relay is $n_{m}$. If $MP\_UE_{n}\left(m_{n}\right)>MP\_UE_{n}\left(l_{n}\right)$, it signifies that the matching priority of the *n*th UE with the $m_{n}$th relay is higher than the matching priority of the *n*th UE with the $l_{n}$th relay. We also define the relay with the highest matching priority of the *n*th UE as $MP\_UE_{n}^{\mathrm{highest}}$. Correspondingly, we define the UE with the highest matching priority of the *m*th relay as $MP\_RE_{m}^{\mathrm{highest}}$. In this paper, to maximize system throughput, we have

(20) $$MP\_UE_{n}^{\mathrm{highest}}=\underset{m}{\arg}\;\;\;\;\;\max_{m\in M_{n}}^{}\;\;\;h_{mn},$$

and

(21) $$MP\_RE_{m}^{\mathrm{highest}}=\underset{n}{\arg}\;\;\max\;\;\;\;R_{mn}.$$

The reason for our choice of the throughput $R_{mn}$ and small-scale channel coefficient $h_{mn}$ as the priority judgment criteria of relays and UEs is that they are our optimization function or one of the parameters of the optimization function, and the results screened by these criteria are more conducive to the maximization of throughput.

With the above illustration, the dynamic grouping matching algorithm between UEs and relays can be described as follows. First, we initialize the matching priority according to the available CSI. Then, we divide the grouping process into two matching processes. The first process is to guarantee that each UE can be served by a relay, and the second process is to group the relays for each UE.

In the first process, each UE requests matching the relay that prioritizes the UE over the other UEs. Then, each relay that has received the matching request from the UEs matches the UE which prioritizes the relay over the other relays, and then, it rejects the other UEs. This process is repeated until all UEs are served by at least one relay.

In the second process, each UE requests matching the unmatched relay that has the highest priority to the UE. Subsequently, the relay that has received matching requests from UEs selects the UE according to its matching priority if the number of relays in a group is below *Q*. When the number of relays in a group is *Q*, we determine whether the UE sending this matching request is more effective for improving the throughput than the other UEs in the group.

If this is the case, then we update the matched pair; otherwise, we reject the matching request. This process is repeated until all the relays are grouped or no UEs request matching with any relays. The details of the dynamic grouping matching algorithm are provided in Algorithm 1.
**Algorithm 1** Dynamic Group Matching Algorithm.1:Initialization: Initialize the matched pairing set of the UEs and relays Φ=∅, the unmatched set of the UEs and relays U_UE, U_RE, and initialize the matching priority sets of the UEs and relays {MP_UE}, {MP_RE} through the Equations (20) and (21)2:**while** U_UE≠∅ **do**3:   Each UE in U_UE requests to match its highest matching priority relay from U_RE according to the matching priority set {MP_UE}4:   **for** relay m = 1, 2,…, M **do**5:     Each relay matches the UE with the highest priority according to the matching priority set of relays {MP_RE} and rejects the other UEs6:     The rejected UEs remove the *m*th relay from its matching priority set {MP_UE}7:     Add the matched paring Φ(m,n) to the set Φ and remove the *m*th relay and *n*th UE from U_RE and U_UE, respectively8:   **end for**9:**end while**10:**while**{MP_UE}≠∅ or U_RE≠∅ **do**11:   Each UE requests to match its highest matching priority relay from U_RE according to the updated set {MP_UE}12:   **for** relay *m* = 1, 2,…, M **do**13:     The *m*th relay makes the following judgment for its highest matching priority UE according to its matching priority set {MP_RE}14:     **if** $\sum_{m=1}^{M}\left|\Phi (m,n)\right|<Q$ **then**15:        Add the matched paring Φ(m,n) to the set Φ and remove the *m*th relay and *n*th UE from U_RE and U_UE, respectively16:     **else**17:        The relay matches with the *n*th UE when there exists a relay that satisfies $\psi_{mn}>\psi_{ln}$ and |Φ(l,n)|=1; then, it updates Φ and removes the *l*th relay into U_RE18:        Otherwise, reject the matching request of the *n*th UE and remove the *m*th relay from its matching priority set {MP_UE}19:     **end if**20:   **end for**21:**end while**

### 4.2. Joint Power and Subcarrier Allocation Algorithm for the Cooperative Network

Given the matching outcome described in Section IV, we propose the joint power and subcarrier allocation algorithm based on the D.C. programming to optimize the system throughput of the cooperative network. We denote the assignment $c_{mn}^{k}$ as $c_{mn}^{k}=\left|\Phi \left(m,n\right)\right|b_{n}^{k}$, where |Φ(m,n)|∈{0,1} denotes the matched pair of the *n*th UE and the *m*th relay, and $b_{n}^{k}\in \left\{0,1\right\}$ denotes whether the *n*th UE is served on the *k*th subcarrier or not. Problem (13) can be rewritten as

(22) $$\begin{array}{cc} \; & \underset{b,\beta ,p,q}{\mathrm{maximize}\;}\;\;\tilde{R}\left(b,\beta ,p,q\right)\;\;\\ \; & s.t.C1,C2,C3,C4,C5, \end{array}$$

where $b\in Z^{NK\times 1}$ collects variables $b_{n}^{k},\forall n,k$.

We combine the mixed integer constraint C1 with constraint C5, as given by

(23) $$\begin{array}{cc} C1^{\prime }(a): & 0⩽b_{n}^{k}\beta_{}^{k}=u_{n}^{k}⩽1,\forall n,k\\ (b): & 0⩽b_{n}^{k}\left(1-\beta_{}^{k}\right)=v_{n}^{k}⩽1,\forall n,k\\ (c): & u_{n}^{k}+v_{n}^{k}\in \left\{0,1\right\},\forall n,k, \end{array}$$

The matching between the UEs and relays in constraint *C*1 is obtained by Algorithm 1. Only $b_{n}^{k}$ remains to be solved in constraint *C*1. The integer constraint $C1^{\prime }c$ is equivalent to the following expression:

(24) $$\begin{array}{cc} C6\;(a): & \sum_{n=1}^{N}\sum_{k=1}^{K}\left(\left(u_{n}^{k}+v_{n}^{k}\right)-{\left(u_{n}^{k}+v_{n}^{k}\right)}^{2}\right)⩽0,\\ (b): & 0⩽u_{n}^{k}+v_{n}^{k}⩽1,\forall n,k. \end{array}$$

Now, the optimization with the integer constraints is transformed to a continuous-value problem. We define $u\in R^{NK\times 1}$, and $v\in R^{NK\times 1}$ to collect the variables $u_{n}^{k}$ and $v_{n}^{k}$, respectively. Problem (22) can be reformulated as:

(25) $$\begin{array}{cc} \; & \underset{u,v,p,q}{\mathrm{minimize}\;}\;\;-\tilde{R}\left(u,p\right)\\ \; & s.t.C1^{\prime }a,C1^{\prime }b,C2,C3,C4,C6. \end{array}$$

According to the theorem of monotone optimization [28], the equivalent problem of (25) can be formed as:

(26) $$\begin{array}{cc} \; & \underset{u,v,p,q}{\mathrm{minimize}\;}\;\;-\tilde{R}\left(u,p\right)+\eta \left\{\sum_{n=1}^{N}\sum_{k=1}^{K}\left(\left(u_{n}^{k}+v_{n}^{k}\right)-{\left(u_{n}^{k}+v_{n}^{k}\right)}^{2}\right)\right\},\\ \; & s.t.C1^{\prime }a,C1^{\prime }b,C2,C3,C4,C6b. \end{array}$$

where η is a sufficiently large penalty factor if $u_{n}^{k}+v_{n}^{k}$ is neither 0 nor 1, and η≫1.

Then, we transform the decoding threshold constraint *C*3 into a maximum interference [32] constraint *C*3’ by

(27) $$\begin{array}{cc} C3^{\prime }\; & \left(a\right):\sum_{l=m+1}^{M}\varpi_{ln}|h_{ln}^{k}{|}^{2}c_{ln}^{k}p_{ln}^{k}⩽\xi_{a},\forall m,n,k\\ \; & \left(b\right):\varpi_{m}|h_{m}^{k}{|}^{2}\sum_{l=1}^{m-1}q_{l}^{k}⩽\xi_{l},\forall m,k. \end{array}$$

The new constraint *C*3′ is a convex set. However, the problem is still a non-convex problem, since neither the objective function nor constraint *C*4 is convex. Nevertheless, the following equivalent form always holds,

(28) $$\begin{array}{cc} \log_{2}\left(1+SINR_{mn}^{A,k}\right) & =\log_{2}\left(\sigma^{2}+\sum_{l=m}^{M}\varpi_{ln}|h_{ln}^{k}{|}^{2}p_{ln}^{k}\right)\\ & -\log{}_{2}\left(\sigma^{2}+\sum_{l=m+1}^{M}\varpi_{ln}|h_{ln}^{k}{|}^{2}p_{ln}^{k}\right)\\ \; & ≜f_{mn}^{A,k}\left(p\right)-g_{mn}^{A,k}\left(p\right). \end{array}$$

Therefore, we derive that

(29) $${\tilde{R}}_{n}^{A}=\sum_{k=1}^{K}\sum_{m=1}^{M}u_{n}^{k}\left(f_{mn}^{A,k}\left(p\right)-g_{mn}^{A,k}\left(p\right)\right)≜F_{n}^{A}\left(u,p\right)-G_{n}^{A}\left(u,p\right).$$

Similarly, we have

(30) $${\tilde{R}}_{n}^{B}=\sum_{k=1}^{K}\sum_{m=1}^{M}v_{n}^{k}\left(f_{m}^{B,k}\left(q\right)-g_{m}^{B,k}\left(q\right)\right)≜F_{n}^{B}\left(v,q\right)-G_{n}^{B}\left(v,q\right),$$

where

(31) $$f_{m}^{B,k}\left(q\right)=\log_{2}\left(\sigma^{2}+\varpi_{m}^{}|h_{m}^{k}{|}^{2}\sum_{l=1}^{m}q_{l}^{k}\right),\mathrm{and}$$

(32) $$g_{m}^{B,k}\left(q\right)=\log_{2}\left(\sigma^{2}+\varpi_{m}^{}|h_{m}^{k}{|}^{2}\sum_{l=1}^{m-1}q_{l}^{k}\right).$$

Then, the non-convex constraint *C*4 can be rewritten as

(33) $$\begin{array}{cc} C4^{\prime }\;\left(a\right): & F_{n}^{A}\left(u,p\right)+G_{n}^{B}\left(v,q\right)-\left(G_{n}^{A}\left(u,p\right)+F_{n}^{B}\left(v,q\right)\right)⩽0\\ (b): & {\hat{R}}_{n,\mathrm{target}}^{}+G_{n}^{A}\left(u,p\right)-F_{n}^{A}\left(u,p\right)⩽0. \end{array}$$

Constraint *C*4′ is the difference of two convex Functions (31)–(33). Additionally, we have

(34) $$\begin{array}{cc} -\tilde{R}\left(u,p\right) & =\sum_{n=1}^{N}-F_{n}^{A}\left(u,p\right)-\sum_{n=1}^{N}-G_{n}^{A}\left(u,p\right)\\ & ≜F_{}^{A}\left(u,p\right)-G_{}^{A}\left(u,p\right). \end{array}$$

Therefore, we can rewrite (26) as

(35) $$\begin{array}{cc} \; & \underset{u,v,p,q}{\mathrm{minimize}\;}\;\;F_{}^{A}\left(u,p\right)-G_{}^{A}\left(u,p\right)+\eta \left(H\left(u,v\right)-M\left(u,v\right)\right)\;\;\;\\ & s.t.C1^{\prime }a,C1^{\prime }b,C2,C3^{\prime },C4^{\prime },C6b, \end{array}$$

where $H\left(u,v\right)=\sum_{n=1}^{N}\sum_{k=1}^{K}\left(u_{n}^{k}+v_{n}^{k}\right)$, $M\left(u,v\right)=\sum_{n=1}^{N}\sum_{k=1}^{K}{\left(u_{n}^{k}+v_{n}^{k}\right)}^{2}$.

Note that $F_{n}^{A}\left(u,p\right)$, $G_{n}^{A}\left(u,p\right)$, $F_{n}^{B}\left(v,q\right)$, $G_{n}^{B}\left(v,q\right)$, H(u,v), and M(u,v) are convex functions. Therefore, problem (35) is a D.C. program. We can implement successive convex approximation to obtain a suboptimal solution of the problem [33,34]. Given the differentiability of the convex functions $F_{n}^{A}\left(u,p\right)$, $G_{n}^{A}\left(u,p\right)$, $F_{n}^{B}\left(v,q\right)$, and M(u,v), for any feasible point $u^{\left(\tau \right)}$, $v^{\left(\tau \right)}$, $p^{\left(\tau \right)}$, and $q^{\left(\tau \right)}$, we have

(36) $$\begin{array}{cc} F_{n}^{A}\left(u,p\right) & ⩾F_{n}^{A}\left(u^{\left(\tau \right)},p^{\left(\tau \right)}\right)+\nabla_{u}F_{n}^{A}\left(u^{\left(\tau \right)},p^{\left(\tau \right)}\right)\left(u-u^{\left(\tau \right)}\right)\\ & +\nabla_{p}F_{n}^{A}\left(u^{\left(\tau \right)},p^{\left(\tau \right)}\right)\left(p-p^{\left(\tau \right)}\right)\\ \; & ≜\nabla_{u^{\left(\tau \right)},p^{\left(\tau \right)}}^{\mathrm{affine}}F_{n}^{A}\left(u,p\right), \end{array}$$

(37) $$\begin{array}{cc} G_{n}^{A}\left(u,p\right) & ⩾G_{n}^{A}\left(u^{\left(\tau \right)},p^{\left(\tau \right)}\right)+\nabla_{u}G_{n}^{A}\left(u^{\left(\tau \right)},p^{\left(\tau \right)}\right)\left(u-u^{\left(\tau \right)}\right)\\ & +\nabla_{p}G_{n}^{A}\left(u^{\left(\tau \right)},p^{\left(\tau \right)}\right)\left(p-p^{\left(\tau \right)}\right)\\ \; & ≜\nabla_{u^{\left(\tau \right)},p^{\left(\tau \right)}}^{\mathrm{affine}}G_{n}^{A}\left(u,p\right), \end{array}$$

(38) $$\begin{array}{cc} F_{n}^{B}\left(v,q\right) & ⩾F_{n}^{B}\left(v^{\left(\tau \right)},q^{\left(\tau \right)}\right)+\nabla_{v}F_{n}^{B}\left(v^{\left(\tau \right)},q^{\left(\tau \right)}\right)\left(v-v^{\left(\tau \right)}\right)\\ & +\nabla_{q}F_{n}^{B}\left(v^{\left(\tau \right)},q^{\left(\tau \right)}\right)\left(q-q^{\left(\tau \right)}\right)\\ \; & ≜\nabla_{v^{\left(\tau \right)},q^{\left(\tau \right)}}^{\mathrm{affine}}F_{n}^{B}\left(v,q\right), \end{array}$$

and

(39) $$\begin{array}{cc} M(u,v) & ⩾M\left(u^{\left(\tau \right)},v^{\left(\tau \right)}\right)+\nabla_{u}M\left(u^{\left(\tau \right)},v^{\left(\tau \right)}\right)\left(u-u^{\left(\tau \right)}\right)\\ & +\nabla_{v}M\left(u^{\left(\tau \right)},v^{\left(\tau \right)}\right)\left(v-v^{\left(\tau \right)}\right)\\ \; & ≜\nabla_{u^{\left(\tau \right)},v^{\left(\tau \right)}}^{\mathrm{affine}}M\left(u,v\right). \end{array}$$

In (36)–(39), $\nabla_{u^{\left(\tau \right)},p^{\left(\tau \right)}}^{\mathrm{affine}}F_{n}^{A}\left(u,p\right)$, $\nabla_{u^{\left(\tau \right)},p^{\left(\tau \right)}}^{\mathrm{affine}}G_{n}^{A}\left(u,p\right)$, $\nabla_{v^{\left(\tau \right)},q^{\left(\tau \right)}}^{\mathrm{affine}}F_{n}^{B}\left(v,q\right)$, and $\nabla_{u^{\left(\tau \right)},v^{\left(\tau \right)}}^{\mathrm{affine}}M\left(u,v\right)$ are affine functions of $F_{n}^{A}\left(u,p\right)$, $G_{n}^{A}\left(u,p\right)$, $F_{n}^{B}\left(v,q\right)$, and M(u,v), respectively. The gradients in the affine functions can be given by

(40) $$\begin{array}{cc} \nabla_{u}F_{n}^{A}\left(u^{\left(\tau \right)},p^{\left(\tau \right)}\right) & \left(u-u^{\left(\tau \right)}\right)\\ & =\sum_{k=1}^{K}\sum_{m=1}^{M}f_{mn}^{A,k}\left(p^{\left(\tau \right)}\right)\left(u_{n}^{k}-u_{n}^{k(\tau )}\right), \end{array}$$

(41) $$\begin{array}{cc} \nabla_{p}F_{n}^{A} & \left(u^{\left(\tau \right)},p^{\left(\tau \right)}\right)\left(p-p^{\left(\tau \right)}\right)\\ & =\sum_{k=1}^{K}\sum_{m=1}^{M}\frac{u_{n}^{k(\tau )}\sum_{q=m}^{M}\varpi_{qn}|h_{qn}^{k}{|}^{2}\left(p_{qn}^{k}-p_{qn}^{k(\tau )}\right)}{(\sigma^{2}+\sum_{q=m}^{M}\varpi_{qn}|h_{qn}^{k}{|}^{2}p_{qn}^{k(\tau )})\ln2}, \end{array}$$

(42) $$\begin{array}{cc} \nabla_{u}G_{n}^{A}\left(u^{\left(\tau \right)},p^{\left(\tau \right)}\right) & \left(u-u^{\left(\tau \right)}\right)\\ & =\sum_{k=1}^{K}\sum_{m=1}^{M}g_{mn}^{A,k}\left(p^{\left(\tau \right)}\right)\left(u_{n}^{k}-u_{n}^{k(\tau )}\right), \end{array}$$

(43) $$\begin{array}{cc} \nabla_{p} & G_{n}^{A}\left(u^{\left(\tau \right)},p^{\left(\tau \right)}\right)\left(p-p^{\left(\tau \right)}\right)\\ & =\sum_{k=1}^{K}\sum_{m=1}^{M}\frac{u_{n}^{k(\tau )}\sum_{q=m+1}^{M}\varpi_{qn}|h_{qn}^{k}{|}^{2}\left(p_{qn}^{k}-p_{qn}^{k(\tau )}\right)}{(\sigma^{2}+\sum_{q=m+1}^{M}\varpi_{qn}|h_{qn}^{k}{|}^{2}p_{qn}^{k(\tau )})\ln2}, \end{array}$$

(44) $$\begin{array}{cc} \nabla_{v}F_{n}^{B}\left(v^{\left(\tau \right)},q^{\left(\tau \right)}\right) & \left(v-v^{\left(\tau \right)}\right)\\ & =\sum_{k=1}^{K}\sum_{m=1}^{M}f_{m}^{B,k}\left(q^{\left(\tau \right)}\right)\left(v_{n}^{k}-v_{n}^{k(\tau )}\right), \end{array}$$

(45) $$\begin{array}{cc} \nabla_{q}F_{n}^{B}\left(v^{\left(\tau \right)},q^{\left(\tau \right)}\right) & \left(q-q^{\left(\tau \right)}\right)\\ & =\sum_{k=1}^{K}\sum_{m=1}^{M}\frac{v_{n}^{k(\tau )}\varpi_{m}^{}|h_{m}^{k}{|}^{2}\sum_{q=1}^{m}\left(p_{q}^{k}-p_{q}^{k(\tau )}\right)}{(\sigma^{2}+\varpi_{m}^{}|h_{m}^{k}{|}^{2}\sum_{q=1}^{m}p_{q}^{k(\tau )})\ln2}, \end{array}$$

(46) $$\begin{array}{cc} \nabla_{u}M\left(u^{\left(\tau \right)},v^{\left(\tau \right)}\right) & \left(u-u^{\left(\tau \right)}\right)\\ & =\sum_{n=1}^{N}\sum_{k=1}^{K}2\left(u_{n}^{k(\tau )}+v_{n}^{k(\tau )}\right)\left(u_{n}^{k}-u_{n}^{k(\tau )}\right), \end{array}$$

and

(47) $$\begin{array}{cc} \nabla_{v}M\left(u^{\left(\tau \right)},v^{\left(\tau \right)}\right) & \left(v-v^{\left(\tau \right)}\right)\\ & =\sum_{n=1}^{N}\sum_{k=1}^{K}2\left(u_{n}^{k(\tau )}+v_{n}^{k(\tau )}\right)\left(v_{n}^{k}-v_{n}^{k(\tau )}\right). \end{array}$$

For a given feasible point $u^{\left(\tau \right)}$, $v^{\left(\tau \right)}$, $p^{\left(\tau \right)}$, and $q^{\left(\tau \right)}$, we can achieve the upper bound of (35) by solving the following convex optimization problem:

(48) $$\begin{array}{cc} \underset{u,v,p,q}{\mathrm{minimize}\;}\; & F_{}^{A}\left(u,p\right)-\nabla_{u^{\left(\tau \right)},p^{\left(\tau \right)}}^{\mathrm{affine}}G_{}^{A}\left(u,p\right)+\eta (H\left(u,v\right)\\ & -\nabla_{u^{\left(\tau \right)},v^{\left(\tau \right)}}^{\mathrm{affine}}M\left(u,v\right))\\ s.t. & C1^{\prime }a,C1^{\prime }b,C2,C3^{\prime },C6b\\ C4^{\prime }\;\left(a\right): & F_{n}^{A}\left(u,p\right)+G_{n}^{B}\left(v,q\right)-\nabla_{u^{\left(\tau \right)},p^{\left(\tau \right)}}^{\mathrm{affine}}G_{n}^{A}\left(u,p\right)\\ & -\nabla_{v^{\left(\tau \right)},q^{\left(\tau \right)}}^{\mathrm{affine}}F_{n}^{B}\left(v,q\right)⩽0\\ (b): & {\hat{R}}_{n,\mathrm{target}}^{}+G_{n}^{A}\left(u,p\right)-\nabla_{u^{\left(\tau \right)},p^{\left(\tau \right)}}^{\mathrm{affine}}F_{n}^{A}\left(u,p\right)⩽0, \end{array}$$

where $\nabla_{u^{\left(\tau \right)},p^{\left(\tau \right)}}^{\mathrm{affine}}G_{}^{A}\left(u,p\right)=\sum_{n=1}^{N}-\nabla_{u^{\left(\tau \right)},p^{\left(\tau \right)}}^{\mathrm{affine}}G_{n}^{A}\left(u,p\right)$.

Generally, the convex problem in (48) can be readily settled by standard convex program solvers, and it can be solved by standard convex programming solvers such as CVX [33]. We propose a successive convex approximation to tighten the upper bound solution in (48) by an iterative algorithm, i.e., Algorithm 2. It can generate a sequence of feasible solutions continuously and achieve a locally optimal solution in polynomial time [34].
**Algorithm 2** Iterative Algorithm for Resource Allocation.1:Initialization: Initialize the maximum number of iterations $T_{\max}$ and set iteration index τ=1; and initialize the current feasible point $u^{\left(1\right)}$, $v^{\left(1\right)}$, $p^{\left(1\right)}$, $q^{\left(1\right)}$ and a penalty factor η≫12:**repeat**3:   Set the variables u, v, p, and q to be solved by the standard convex program solvers4:   Evaluate the convex functions $F_{n}^{A}\left(u,p\right)$, $G_{n}^{A}\left(u,p\right)$, $G_{n}^{B}\left(v,q\right)$, and H(u,v)5:   According to the current point $u^{\left(\tau \right)}$, $v^{\left(\tau \right)}$, $p^{\left(\tau \right)}$, and $q^{\left(\tau \right)}$, evaluate the affine functions $\nabla_{u^{\left(\tau \right)},p^{\left(\tau \right)}}^{\mathrm{affine}}F_{n}^{A}\left(u,p\right)$, $\nabla_{u^{\left(\tau \right)},p^{\left(\tau \right)}}^{\mathrm{affine}}G_{n}^{A}\left(u,p\right)$, $\nabla_{v^{\left(\tau \right)},q^{\left(\tau \right)}}^{\mathrm{affine}}F_{n}^{B}\left(v,q\right)$, and $\nabla_{u^{\left(\tau \right)},v^{\left(\tau \right)}}^{\mathrm{affine}}M\left(u,v\right)$6:   Substitute them into (48) to solve the convex problem for getting the upper-bound optimal point $u^{\mathrm{opt}}$, $v^{\mathrm{opt}}$, $p^{\mathrm{opt}}$, and $q^{\mathrm{opt}}$7:   Update iteration index τ=τ+18:   Update the next iteration point $u^{\left(\tau \right)}=u^{\mathrm{opt}}$, $v^{\left(\tau \right)}=v^{\mathrm{opt}}$, $p^{\left(\tau \right)}=p^{\mathrm{opt}}$, $q^{\left(\tau \right)}=q^{\mathrm{opt}}$9:**until**$\left(u^{\left(\tau -1\right)},v^{\left(\tau -1\right)},p^{\left(\tau -1\right)},q^{\left(\tau -1\right)}\right)=\left(u^{\left(\tau \right)},v^{\left(\tau \right)},p^{\left(\tau \right)},q^{\left(\tau \right)}\right)$ or $\tau =T_{\max}$10:Output the suboptimal solution $u^{*}=u^{\left(\tau \right)}$, $v^{*}=v^{\left(\tau \right)}$, $p^{*}=p^{\left(\tau \right)}$, $q^{*}=q^{\left(\tau \right)}$

### 4.3. Computational Complexity Analysis

The computational complexity of an exhaustive search in the grouping matching algorithm is $O(2^{M}N)$. The exhaustive search scheme is user-centric, as it divides each UE into a group, and each relay can either belong to the group of the UE or not. Thus, the solution to all groupings is $2^{M}N$, and the computational complexity of the exhaustive search is $O(2^{M}N)$. The computational complexity of Algorithm 1 is $O(NM^{2})$. Specifically, NM steps are needed, while each UE matches with a relay for grouping in the proposed grouping algorithm, and the steps for the grouping process are less than M·NM steps. Therefore, the total computational complexity of the proposed grouping algorithm is $O(NM^{2})$. The computational complexity of the D.C. programming is $O(T_{\max}M)$; as $T_{\max}$ is no more than QM, the computational complexity of the D.C. programming is O(M). Thus, the computational complexity is $O(NM^{3})$.

### 4.4. Convergence of Algorithm 1

We divide the algorithm into two processes, and the first process guarantees UE communications. The system performance is slightly degraded to satisfy QoS. The second process of the algorithm is convergent, and the proof is as follows.

**Proof.**  When UE $n_{a}$ matches with relays $m_{2}$, $m_{3}$, …, and $m_{Q}$ (the descending order of priority) in the second process, assuming that there exists a relay $m_{q}$ matching UE $n_{b}$, the priority of $m_{q}$ for $n_{a}$ is higher than that of $m_{Q}$. $n_{a}$ is higher than $n_{b}$ in the priority list of relay $m_{q}$ simultaneously. □

When UE $n_{a}$ matches relay $m_{Q}$ not $m_{q}$, there are two situations: UE $n_{a}$ sends a request to $m_{q}$, and relay $m_{q}$ is rejected, which indicates that the priority of $n_{b}$ is higher than $n_{a}$ in the priority list of $m_{q}$. $n_{a}$ does not send the request to $m_{q}$. We can conclude that the priority of $m_{Q}$ is higher than $m_{q}$ in the priority list of $n_{a}$. The two situations of the hypothesis cannot exist simultaneously, and thus, the hypothesis is not true. There are no better matched pairs, so the matched pair obtained is stable.

## 5. Simulation Results and Analysis

**Figure 2:**
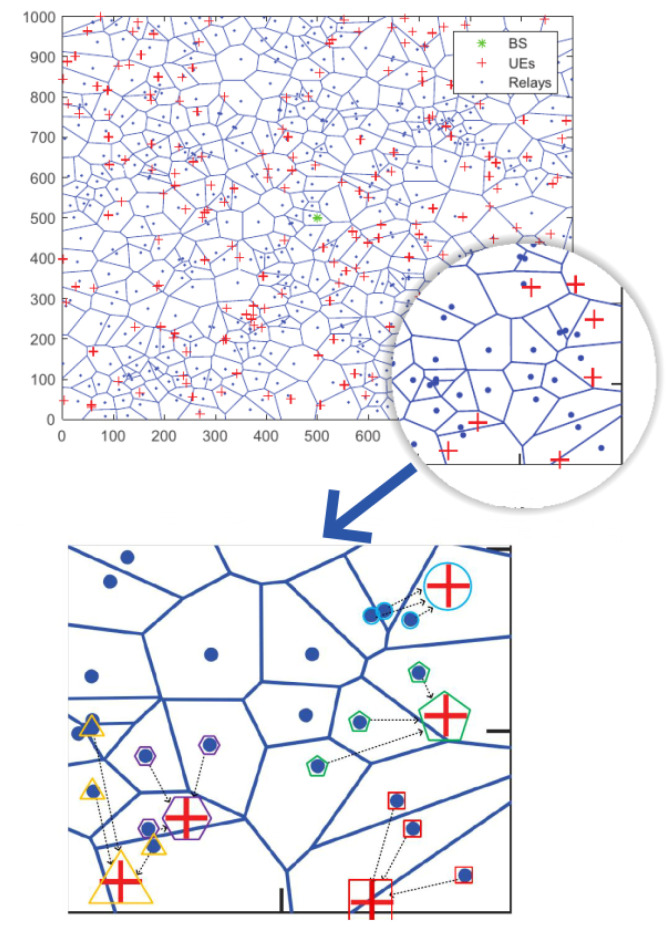
The deployment of BS, UEs, and relays in a 1000 m × 1000 m area. The cell boundaries form a Voronoi tessellation.

In this section, we evaluate the proposed framework and algorithms in terms of the system throughput through simulations. To make a fair comparison, we try to use the same system configuration in OMA, Co-OMA and the traditional NOMA system with the proposed scheme. We deploy the BS in the middle of a 1000 m × 1000 m area. The UEs and relays are modeled as independent PPP with density $\lambda_{u}$ and $\lambda_{r}$. UEs are generated in the area randomly, as illustrated in the top of Figure 2. Other simulation parameters are summarized in Table 2. To show the grouping directly, we provide the schematic diagram when the maximum stack semaphore Q in our proposed scheme is set as 3. The matched pairs of relays and UEs chosen by our proposed algorithms are shown at the bottom of Figure 2. The codes are developed on MATLAB using the CVX toolbox and are executed on a 64-bit operating system with 16 GB RAM and Intel CORE i7, 3.4 GHz.

**Figure 3:**
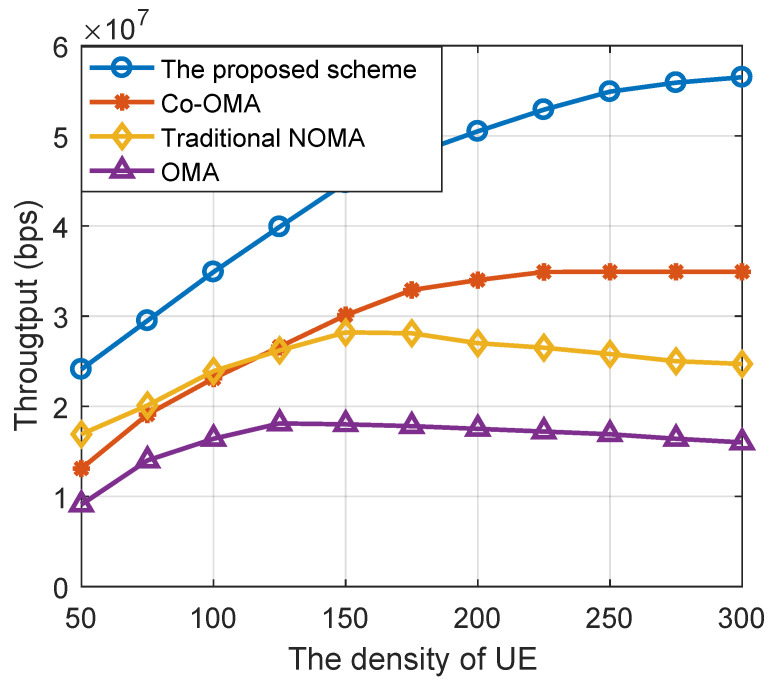
The system throughput versus the density of UEs for different schemes.

In Figure 3, we compared the system throughput of the proposed NOMA-based cooperative network scheme, the OMA-based cooperative network (Co-OMA) scheme, the NOMA, and the OMA scheme under different densities of UEs. The density of relays was set to 300. According to Figure 3, we can see that the proposed scheme achieved the highest system throughput, and it exhibited a 50% gain when the density of UEs exceeded 200 compared to the Co-OMA scheme. This is because with the increasing density of UEs, the spectrum resources is limited, and the proposed scheme shows the advantage of using non-orthogonal resources.

In addition, NOMA and OMA solutions without collaborative communication technology can provide high overall throughput when the density of user nodes becomes too high. This is because to ensure the QoS of some weak channel UEs, a large number of system resources are sacrificed to the specific UEs, which leads to the slow decline of the total throughput of the system and eventually tends to be stable. The comparison between NOMA and cooperative OMA in [35] shows the relationship between backhaul capacity and micro-area access number. When the number of UEs served by the system exceeds the threshold value, the system performance will decline OMA with sufficient system resources (low user node density). However, the user node density increases, and the cooperative OMA can improve the throughput by using the channel gain of the backhaul link and the multiplexing gain of a large number of relay nodes, thus exceeding the throughput performance of a non-collaborative NOMA scenario.

To verify the effectiveness of our proposed algorithms, we compared it with the following benchmarks in the NOMA-based cooperative network: (1) opportunistic with DCP-based allocation (ORG-DCPA), (2) dynamic relay grouping with fixed transmit power allocation (DRG-FTPA), and (3) opportunistic relay grouping with fixed transmit power allocation (ORG-FTPA). The four algorithms can be seen as the combination of DRG, ORG, DCPA and FTPA. The computational complexity of DRG is $O(N\cdot M^{2})$ as shown in the analyses above; the worst case of ORG is exhaustive search, so the computational complexity is $O(N\cdot 2^{M})$. The computational complexity of DCPA and FTPA are O(M) and O(1), respectively. Thus, the complexity of DRG-DCPA, ORG-DCPA, DRG-FTPA, and ORG-FTPA can be present as $O(N\cdot M^{3})$, $O(M\cdot N\cdot 2^{M})$, $O(N\cdot M^{2})$, and $O(N\cdot 2^{M})$. It needs to be emphasized that although DRG-FTPA has the lowest complexity, due to the unchangeable natuer of the transmit power, the DRG-FTPA shows the worst performance during the simulation. Then, we showed the simulation results in terms of the maximum power of relays, the density of relays and the density of UEs.

**Figure 4:**
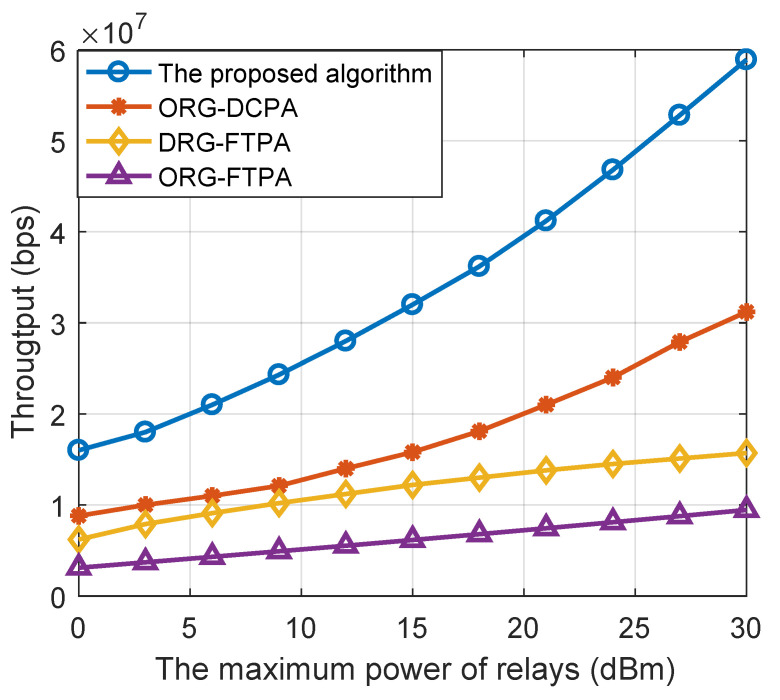
System throughput versus the maximum power of the relays with different resource allocation algorithms.

Figure 4 shows the system throughput diagram of several algorithm schemes under different relay transmitting powers, where the densities of UEs and relays are 200 and 500, respectively. The proposed DRG-DCPA resource allocation algorithm can obtain the highest system throughput, which is followed by the ORG-DCPA algorithm and ORG-FTPA algorithm. In particular, compared with the system throughput of other resource allocation algorithms, the DRG-DCPA algorithm can at least double the system throughput when the maximum power is more than 15 dBm.

**Figure 5:**
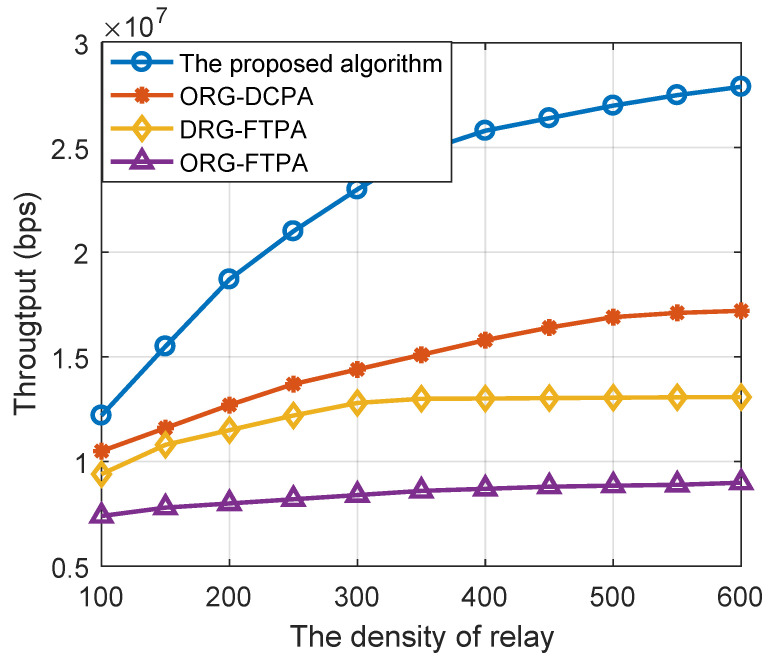
The system throughput versus the density of relays for using different optimization algorithms.

Figure 5 shows the system throughput comparison of the above resource allocation algorithm schemes under different relay densities where the user node density is set to 50. When the density of relays is large enough, the system throughput of the DRG-DCPA resource allocation algorithm improved by more than 60%, 100% and 200%. When the density of intermediate relays increases, the system throughput of the DRG-DCPA algorithm improves faster, indicating that the algorithm has a stronger ability to utilize relay resources and thus can obtain more multiplexing gain.

**Figure 6:**
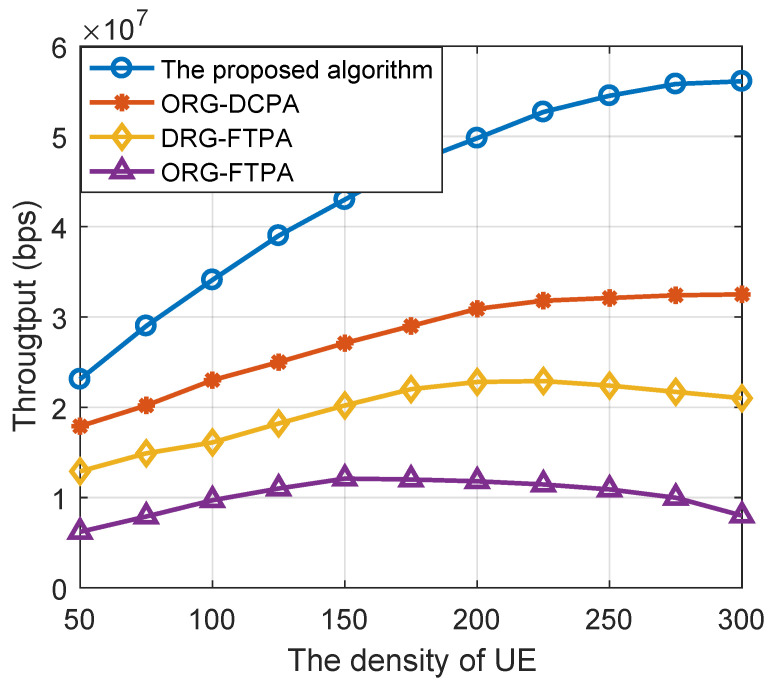
The system throughput versus the density of UEs for using different optimization algorithms.

Figure 6 shows the system throughput comparison of the above resource allocation algorithm schemes for different densities of UE. The density of the relay is set to 300. When the relay node density is sufficient, the throughput of the DRG-DCPA algorithm will increase with increasing user density, and when the user node density is 200, the throughput of the DRG-DCPA algorithm will increase by more than 60%, 120% and 210% compared with the other three algorithms. In addition, the use of a fixed percentage of the power allocation algorithm under the condition of excessive user node density decreases. This is because the above allocation algorithm may spend too much power to weak channel users to ensure the QoS. This causes the system to not effectively utilize resources; when the user node is too saturated, the system throughput will deduce.

**Figure 7:**
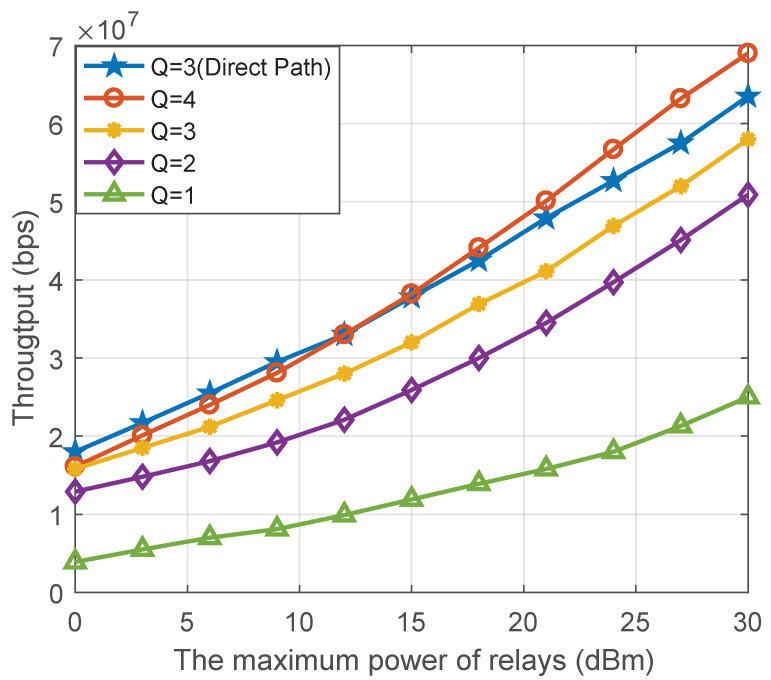
The system throughput versus the maximum power of the relays with different maximum numbers of superposed signals.

In Figure 7, we present the effect in the proposed collaborative system on throughput with different maximum numbers of signals superposed per subcarrier. When the maximum relay power is 15 dBm, the proposed system can improve the throughput more than twice under QoS constraints. In addition, we use Q to denote the maximum number of signals superposed per subcarrier; when Q is larger, the proposed scheme can achieve a higher throughput. In particular, the throughput increases rapidly when the intermediate maximum power is higher. One possible reason is that the number of overstacked signals at low SINR will make the interference in the SIC decoder too large to meet the constraint *C*3; thus, the gain of the throughput is reduced.

It should be noted that the influence of the direct path is not considered in the reference, and the channel condition is poor. The simulation comparison result is the lower bound of the system and algorithm performance, but it can also effectively demonstrate the effectiveness of the proposed collaborative system and optimization algorithm. To confirm the above conjecture, we give a further simulation. In this simulation, we consider the direst path scenario. In addition, if the impact of the direct path on the system is considered, there is generally a direct path between the BS and the relay, but there is no direct path between the relay and the user. At this point, the channel between the base station and the relay is better, which is generally regarded as the Rician fading channel, and the improvement of the channel condition can help relax the constraint *C*3. The simulation shows that in the low SINR, Q = 3 with direct path performances better than Q = 4, but as the power increased, the Q = 4 without a direct solution plays a non-orthogonal higher resource utilization. The simulation result can further improve our conjecture.

## 6. Conclusions

In this paper, the NOMA-based multi-user and multi-relay cooperative network has been studied. To maximize the system throughput, we have designed the resource allocation algorithm as a mixed integer non-linear programming problem. To improve its tractability, we have divided the problem between (1) dynamic group matching of relays and UEs and (2) DCP-based joint allocation of power and subcarriers. Simulation results have confirmed that higher system throughput can be achieved through the proposed algorithm. Compared with Co-OMA, OMA, and NOMA, the proposed algorithm had the highest throughput. The proposed algorithm can also increase the system throughput substantially when the maximum power of the relays is high. The superiority of the proposed algorithm was substantiated by comparing it with different algorithms under various user density and relay density configurations. Simulation results confirmed that the proposed algorithms can be appropriately applied to IoT scenarios with massively small UEs.

## Figures and Tables

**Table 1:** Notations of symbols.

Symbols	Description
$\lambda_{u}$	The density of UE
$\lambda_{r}$	The density of relay
α	Path loss exponent
$\beta^{k}$	The proportion of a time slot occupied by the access stage on the *k*th subcarrier
$x_{mn}^{k}$	Signal transmitted from the *m*th relay to the *n*th UE on the *k*th subcarrier
$p_{mn}^{k}$	Transit power of the signal $x_{mn}^{k}$
$h_{mn}^{k}$	Small-scale fading channel coefficients of $x_{mn}^{k}$
$\varpi_{mn}^{}$	Large-scale fading channel coefficients of $x_{mn}^{k}$
$y_{n}^{A,k}$	Signal received on the *k*th subcarrier of the *n*th UE
$x_{m}^{k}$	Signal transmitted from the BS to the *m*th relay on the *k*th subcarrier
$q_{m}^{k}$	The power of $x_{m}^{k}$
$h_{m}^{k}$	Small-scale fading channel coefficients of $x_{m}^{k}$
$\varpi_{m}$	Large-scale fading channel coefficients of $x_{m}^{k}$
$y_{m}^{B,k}$	Signal received on the *k*th subcarrier of the *m*th relay
$SINR_{mn}^{A,k}$	The received SINR of the *n*th UE served by the *m*th relay on the *k*th subcarrier
${\tilde{R}}_{n}^{A}$	The forward throughput of the *n*th UE
$SINR_{m}^{B,k}$	The signal-to-interference-plus-noise ratio (SINR) of the *m*th relay on the *k*th subcarrier
${\tilde{R}}_{n}^{B}$	The backhaul throughput for the *n*th UE
$\tilde{R}$	The whole system throughput

**Table 2:** Parameters setting.

Parameters	Value
The system bandwidth	3 MHz
Carrier frequency	2 GHz
The subcarrier bandwidth	15 KHz
Path loss exponent α	3.5
Noise power spectral density	−174 dBm/Hz
The density of relays $\lambda_{r}$	500 per/km^2^
The density of UEs $\lambda_{u}$	200 per/km^2^
The maximum power of BS	46 dBm
The maximum power of relays	0:3:30 dBm
The target data rate of UEs	2 bps/Hz

## Data Availability

No new data were created or analyzed in this study. Data sharing is not applicable to this article.

## Abbreviations

The following abbreviations are used in this manuscript:

NOMA	Non-orthogonal multiple access
IoT	Internet of things
OMA	Orthogonal multiple access
SCMA	Sparse code multiple access
PDMA	Pattern division multiple access
MIMO	Multiple input multiple output
DCP	Difference of convex functions programming
QoS	Quality of service
BS	Base station
UE	User equipment
PPPs	Poisson point processes
TDD	Time division duplex
AWGN	Additive white Gaussian noise
SIC	Successive interference cancellation
SINR	Signal-to-interference-plus-noise ratio
Co-OMA	OMA-based cooperative network
ORG-DCPA	Opportunistic with DCP-based allocation
DRG-FTPA	Dynamic relay grouping with fixed transmit power allocation
ORG-FTPA	Opportunistic relay grouping with fixed transmit power allocation
